# China’s most typical nonferrous organic-metal facilities own specific microbial communities

**DOI:** 10.1038/s41598-018-30519-1

**Published:** 2018-08-22

**Authors:** Jian-li Liu, Jun Yao, Fei Wang, Wen Ni, Xing-yu Liu, Geoffrey Sunahara, Robert Duran, Gyozo Jordan, Karen A. Hudson-Edwards, Lena Alakangas, Tatjana Solevic-Knudsen, Xiao-zhe Zhu, Yi-yue Zhang, Zi-fu Li

**Affiliations:** 1 School of Energy and Environment Engineering, University of Science and Technology Beijing, Beijing, 100083 China; 2 School of Water Resource and Environment Engineering, China University of Geosciences, Beijing, 100083 China; 3 School of Civil and Resource Engineering, University of Science and Technology Beijing, Beijing, 100083 China; 4 Gen Res Inst Nonferrous Met, Natl Engn Lab Biohydromet, Beijing, 100088 China; 5 Department of Natural Resource Sciences, McGill University, Montreal, Quebec, H9X3V9 Canada; 6 Equipe Environnement et Microbiologie, MELODY group, Université de Pau et des Pays de l’Adour, IPREM UMR CNRS 5254, BP 1155, 64013 Pau Cedex, France; 7 Department of Applied Chemistry, Szent István University, Villányi út 35-43, 1118 Budapest, Hungary; 8 Environment & Sustainability Institute and Camborne School of Mines, University of Exeter, Penryn, Cornwall TR10 9DF UK; 9 Department of Chemical Engineering and Geosciences, Luleå University of Technology, SE-97187 Luleå, Sweden; 10 Institute of Chemistry, Technology and Metallurgy, University of Belgrade, Njegoseva 12, POBox 473, 11001 Belgrade, Serbia

## Abstract

The diversity and function of microorganisms have yet to be explored at non-ferrous metal mining facilities (NMMFs), which are the world’s largest and potentially most toxic sources of co-existing metal(loid)s and flotation reagents (FRs). The diversity and inferred functions of different bacterial communities inhabiting two types of sites (active and abandoned) in Guangxi province (China) were investigated for the first time. Here we show that the structure and diversity of bacteria correlated with the types of mine sites, metal(loid)s, and FRs concentrations; and best correlated with the combination of pH, Cu, Pb, and Mn. Combined microbial coenobium may play a pivotal role in NMMFs microbial life. *Arenimonas*, specific in active mine sites and an acidophilic bacterium, carries functions able to cope with the extreme conditions, whereas Latescibacteria specific in abandoned sites can degrade organics. Such a bacterial consortium provides new insights to develop cost-effective remediation strategies of co-contaminated sites that currently remain intractable for bioremediation.

## Introduction

Environmental co-contamination of metal(loid)s and flotation reagents (FRs) at non-ferrous metal mining facilities (NMMFs) is a global and continuing issue^1–3^. In China, the discharge of metal(loid)s-bearing mine tailings sites accounted for 42% of total industrial emissions in 2013^4^, and FRs were three times more enriched than pesticides in agricultural fields^5^. NMMFs in China represent a large and dangerous source of contamination. Currently, the frequencies of NMMFs pollution incidents threaten the safety of water, soil, and ecologically sensitive environments, which can lead to devastating human health and socio-economic consequences^6,7^. The geochemical characteristics of tailings are modified through time by biotic and abiotic process^8^. Although FRs at NMMFs can be degraded with time^9^, complexation reactions between metal(loid)s and intermediate metabolites of FRs could result in the migration and transformation of metal(loid)s that are more toxic and mobile than the parent compounds^10,11^. These reactions make metal(loid)s easier to migrate and enter the biological chain, leading to aggravated, complex, and regionalized pollution. Therefore, remediation of NMMF sites is an urgent environmental issue around the world^7^. Microorganisms play a key role in biogeochemical cycles, particularly in mine tailings where they are responsible for metal(loid)s transformations under harsh conditions^12–15^. Understanding the organization of microbial communities and their modification through time will provide new insights on how microorganisms colonize such adverse habitats, providing useful information for the implementation of remediation strategies.

Guangxi, a most typical nonferrous metal mining, mineral, and smelting district in China, hosts the largest NMMF sites in Asia comprising active and abandoned tailings^3^. Here we refer to two types of NMMFs sites: ‘active sites’ are those in use for waste management by an owner (operator), whereas ‘abandoned sites’ are facilities having no identified former owner/licensee or not having been closed in a regulated manner^16^. Guangxi province, an ecologically fragile region located in the Pearl River source and has a karst landscape, is thus threatened by mine waste deposits over several decades^17^. Only few studies have investigated Guangxi’s mine tailings^18–20^, so information on microbial communities inhabiting the tailings is scarce. The possibility to compare microbial communities inhabiting active and abandoned nonferrous tailings sites will allow determination of the geochemical factors driving the organization of microbial assemblages. The objectives of the present study were to: (1) determine the bacterial community composition by 16S rRNA gene MiSeq Illumina sequencing at active and abandoned Guangxi’s mine tailings with diverse geochemical characteristics, (2) compare the bacterial communities to define the specific bacterial populations and their functional profiles, and (3) identify the driving factors governing bacterial community organization by correlating bacterial diversity data with geochemical characteristics.

## Results and Discussion

**Figure 1:**
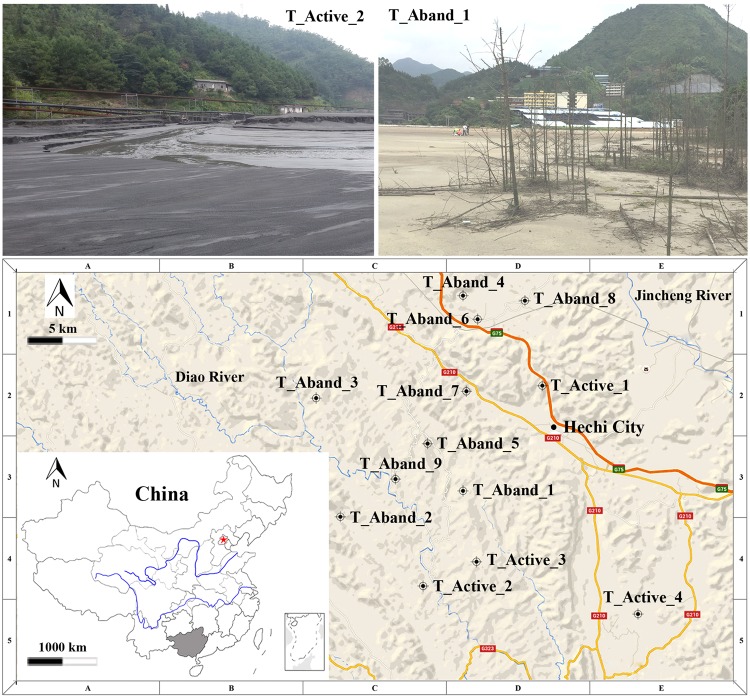
Geographic locations of the 13 NMMFs sites in Guangxi (China), where the surface samples were collected from active waste sites ( T_Active) and abandoned waste sites ( T_Aband). Inset map shows Jinchenjiang District (gray, center of inset), where locations of Guangxi province. Beijing city is denoted by red star ().

Samples were collected from four active and nine abandoned tailings sites (Fig. 1). The distributions of FRs, pH, TOC, TN, TP, and metal(loid)s at the study sites are shown in Fig. S1, Table S1, and Table S2. The average metal(loid)s contents exceeded, and the total C/N/P contents were lower, than the average background soil levels of China^21^, suggesting that the NMMFs sites were nutrient poor or even oligotrophic. Except for Sb, no significant differences (*p* > 0.05) in geochemical parameters were found between active and abandoned sites (Table S3).

Subsamples of each site were also combined (composite samples) and analyzed for bacterial community composition by 16S rRNA MiSeq sequencing. The active sites had a high bacteria diversity (average Shannon index 4.27; Table S4), similar to earlier reports of Pb-Zn mining sites^22,23^. The richness of bacteria communities in active and abandoned sites was similar to those in Pb-Zn, acidic, and chlor-alkali mine sites, but different compared to a rare earth element (REE) mine^21–25^. In all composite samples, α-diversity indices were significantly correlated with the combination of pH, Cu, Pb, and Mn (*r* = 0.53–0.59, *p* < 0.05; Table S5), indicating that the bacterial community structures were mainly driven by geochemical factors, excluding FRs. This has been confirmed by CCA analysis (Fig. S2). The bacterial communities in the tailings sites were distributed within three clusters (Fig. S2): i) bacterial communities marginally correlated with pH and Mn (*r* = 0.24–0.35, 0.08 < *p* < 0.28), ii) bacterial communities significantly correlated with Cu (*r* = 0.45, *p* < 0.05), and iii) bacterial communities significantly correlated with Pb (*r* = 0.80, *p* < 0.05, Fig. S2b and Table S6). As well, water and oxygen favor microbial activities that can result in the production of acidic mine drainage where important metal transformation occurs^26^. Therefore, the acidity of the studied tailings sites (average pH = 5.2, Table S2) may also correlate with the presence of water and oxygen. Although Sb content was significantly different in active and abandoned sites (*t -*value = −2.91; *p* = 0.01; Table S2), the combined influence of these geochemical factors on bacteria communities was more statistically significant than the individual or other remaining combinations, especially for the combination of metal(loid)s and FRs (Table S5).

**Figure 2:**
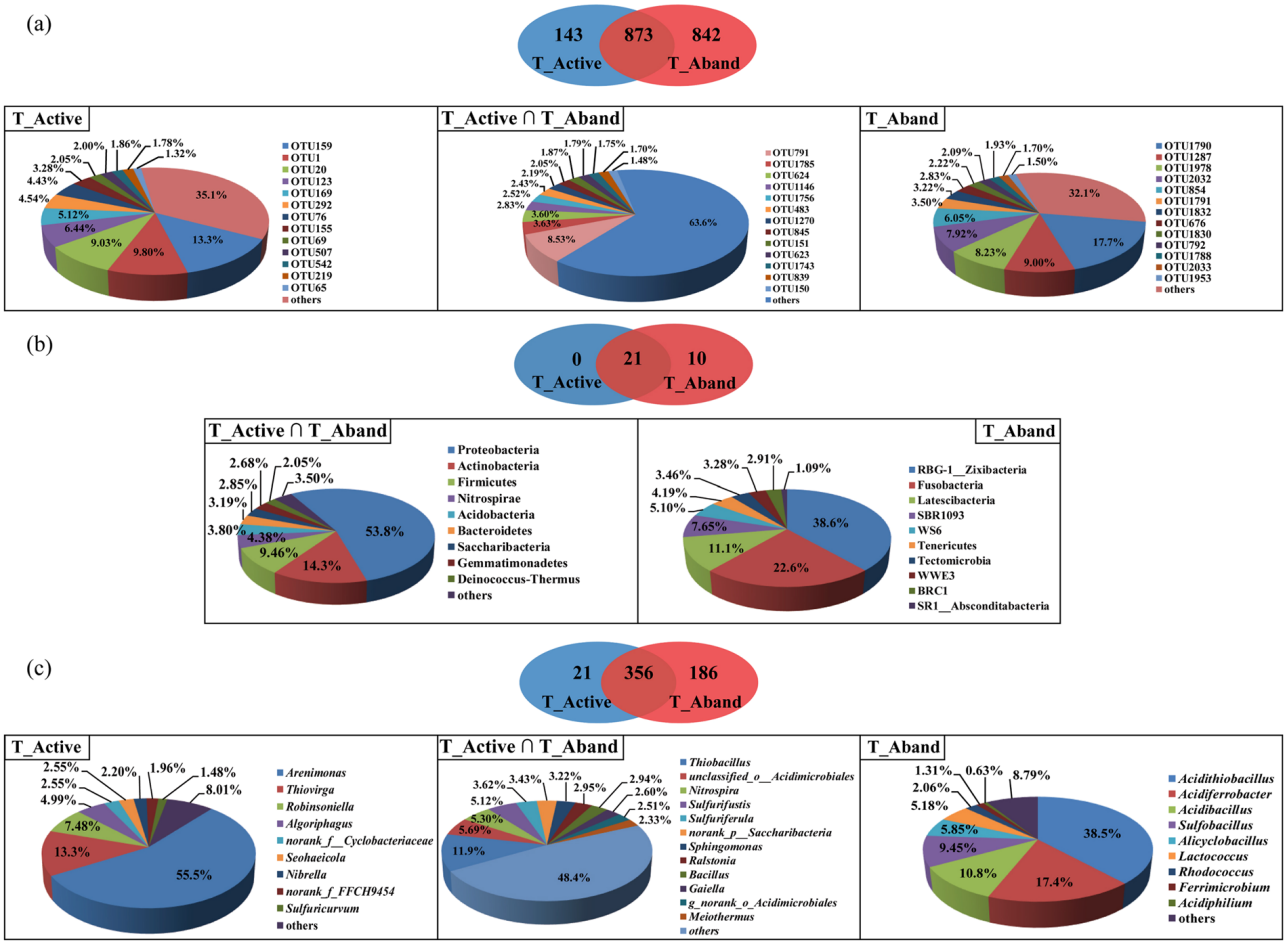
Venn diagram (center) showing the degree of overlap of bacteria between active waste sites (T_Active) and abandoned waste sites (T_Aband) based on OTUs (**a**), phylum (**b**) and genus (**c**) levels. The pie plots show the relative abundance of bacterial species (>1%). The active and abandoned sites share 873 OTUs and 356 genera (T_Active ∩ T_Aband).

**Figure 3:**
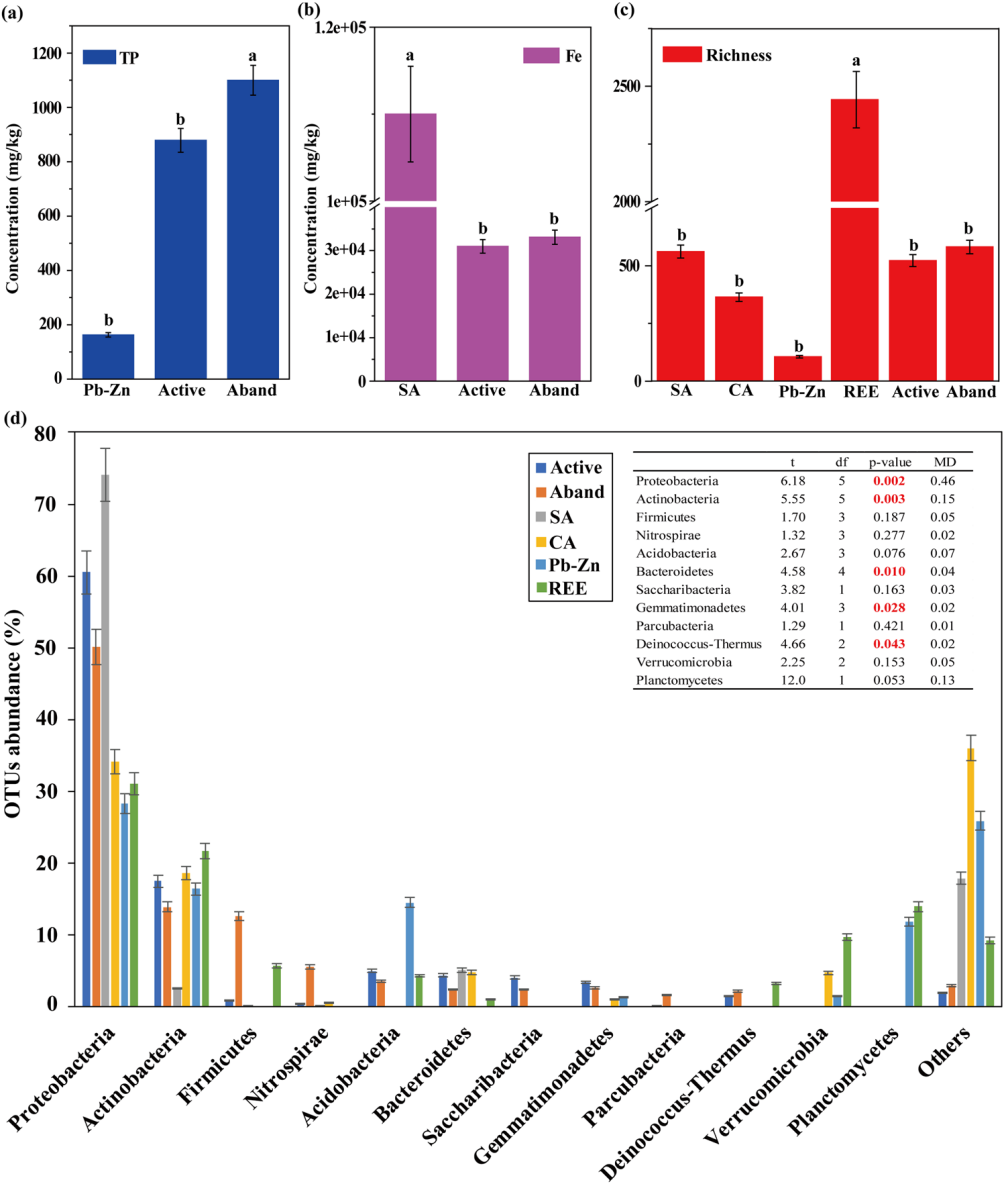
(**a–c**) Meta-analyses of differences of geochemical factors and richness of bacterial communities (only the sites with significant differences using ANOVA are shown). (**d**) Abundance (%) of bacterial communities in different type of sites at the phylum level. Active: active sites in present study; Aband: abandoned sites in present study; SA, slightly acidic sites22; Pb-Zn, an abandoned Pb-Zn mine^23^, REE: a rare Earth element mine^24^; CA, Chlor-alkali sites^25^. The inset Table in Fig. 3d shows statistical results of *t*-test using the independent samples. df: degrees of freedom, MD: Mean Difference. Significant differences at *p* ≤ 0.05 are shown in bolded red text. Other data in black un-bolded text represent non-significant differences at *p* > 0.05.

More than 47% of the total operational taxonomic units (OTUs) were shared between active and abandoned sites, and were associated with Proteobacteria, Firmicutes, Nitrospirae, Actinobacteria, and Acidobacteria (Fig. 2). Our results are confirmed earlier studies^21–25^ and indicated that the bacterial communities inhabiting Guangxi NMMFs area are original. Although mine sites of the present and earlier studies^21–25^ at different geographic locations had a similar structure of dominant bacteria communities, the relative abundances of Proteobacteria, Actinobacteria, and Bacteroidetes were significantly different (*p* < 0.05) (see meta-analyses in Fig. 3d). Results of ANOVA using the data from these sites (active, abandoned, and literature^21,24^ also indicated that TP and Fe had significant differences (*p* < 0.05, Fig. 3). The observation that the bacterial communities are correlated with the type of tailings emphasizes that a successful bioremediation technology should be specific to the type of tailings.

**Figure 4:**
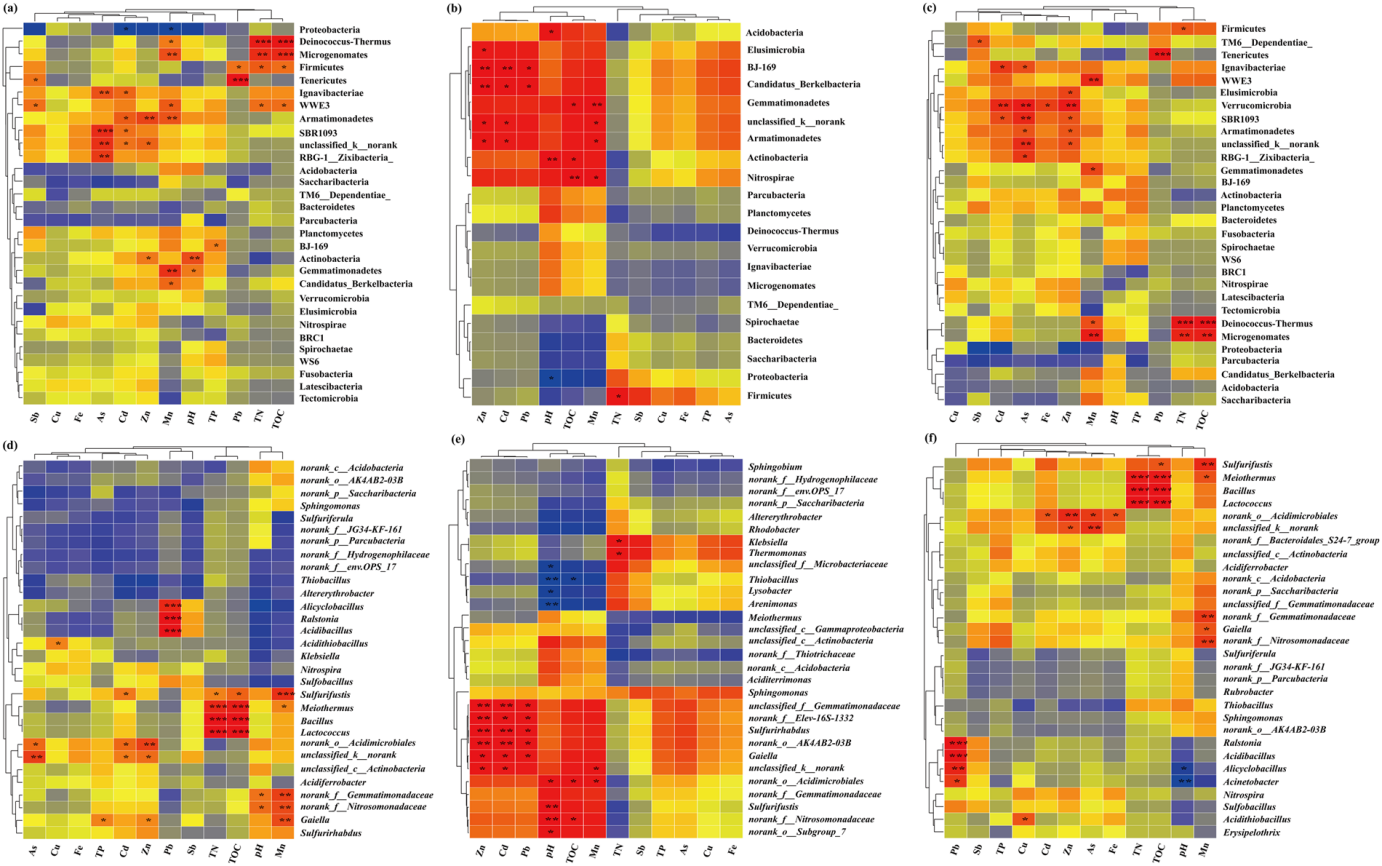
Bacterial-geochemical properties relationships for 13 sites (left panel), active waste sites (middle panel) and abandoned waste sites (right panel) at the phylum (**a–c**) and genus (**d–f**) levels. Heatmaps of the Pearson correlation coefficient values were calculated for bacterial communities (horizontal) and geochemical parameters (vertical) of NMMFs samples, using relative microbial abundance data (ranking the top 30 species). Dendrograms of hierarchical cluster analysis are shown on the left of each heatmap. Color key for the correlation values is shown on the right panel inset; positive correlations are in red text, negative correlations are in blue, non-significant correlations are shown in yellow. *0.01 < *p* ≤ 0.05; **0.001 < *p* ≤ 0.01; ***p ≤ 0.001.

**Figure 5:**
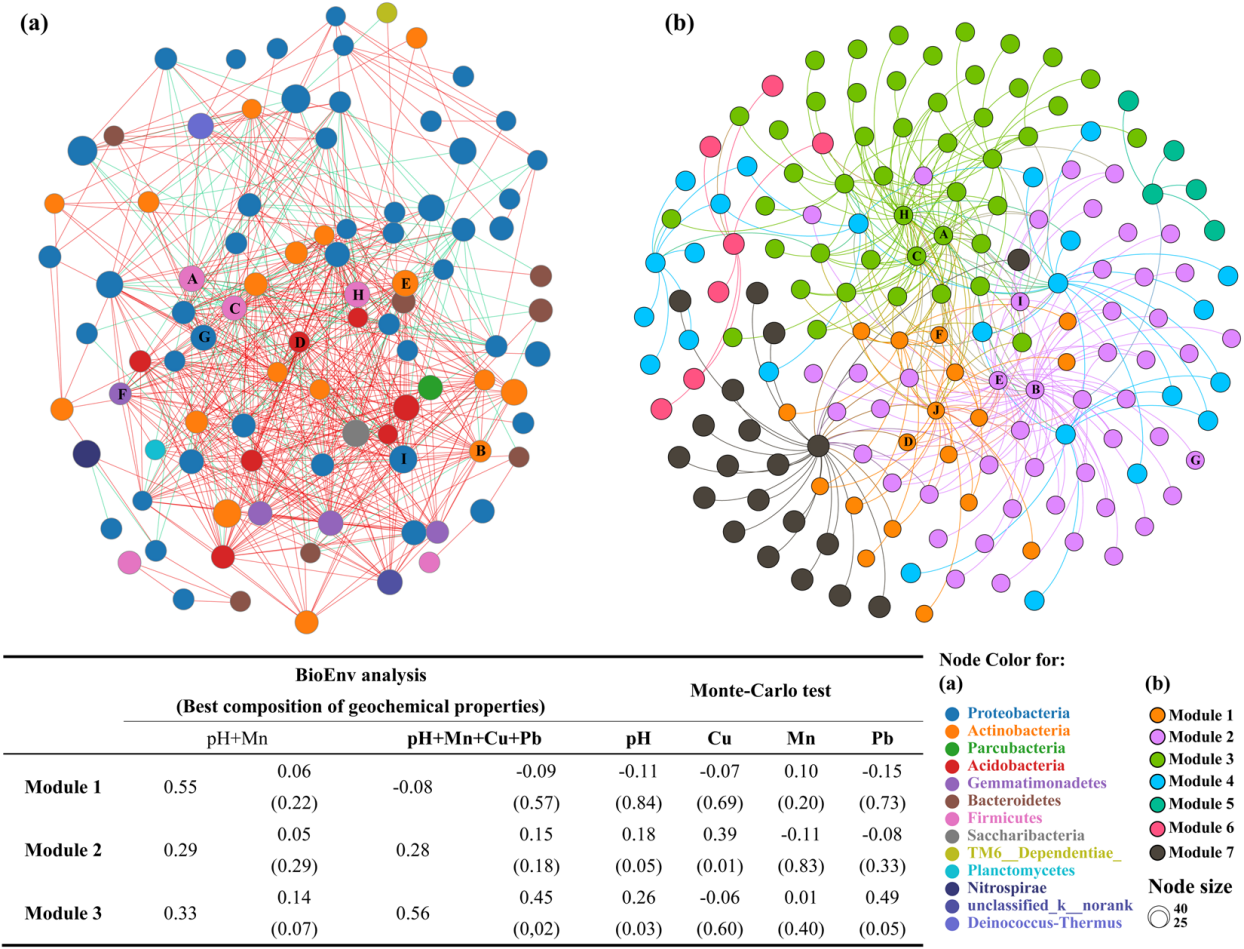
(**a**) Network analysis of overall species correlations between active and abandoned sites based on the most abundant genera. Top 100 was used for analysis. Each node indicates one genus. Colors of node represent the different major phyla. The size of the species-node denotes abundance of species. The red line represents the positive correlation between the two individual nodes, the green line shows a negative (exclusion) correlation. The degree of node was assessed by the numbers of nodes connected directly to that node. The more lines on the node, the higher is the degree, which indicates the intensity of correlation between the species and other species. (**b**) The sub-network analysis for modularity of genera. Colors of node represent the different module. Node size is proportional to the modularity class. The correlation between modules and geochemical properties is shown in inset Table (left bottom). Only modules related to the top 10 high degree genera and best composition of geochemical properties are shown in the correlation analysis. A: *Acidibacillus*, B: *Aciditerrimonas*, C: *Alicyclobacillus*, D: *Bryobacter*, E: *Gaiella*, F: *Gemmatimona*s, G: *Haliangium*, H: *Sulfobacillus*, I: *Sulfurifustis*, and J: *Sulfurirhabdus*.

Sequences related to Proteobacteria were affiliated to eight classes, with *Gammaproteobacteria* being the most abundant and diverse (Fig. S3 and Table S7). In addition, the distributions of Proteobacteria were negatively correlated with Cd and Mn (*r* = −0.56; *p* < 0.05; Fig. 4a). Firmicutes representing 9% (Fig. S3) had diverse ecological optima under the adverse conditions of the present sites, likely by sporulation, and metabolic adaptation^27^. These bacteria were significantly correlated with TN, TOC, and Pb contents (*r* = 0.60–0.67; *p* < 0.05; Fig. 4a). Among the 873-shared OTUs (Fig. 2), *Thiobacillus* were the dominant genera (Fig. 2b), and showed significant differences between active and abandoned sites (*p* = 0.04; Fig. S4); notable significant correlations were found with pH and TOC at active sites (*p* < 0.05; Fig. 4e). *Thiobacillus* are capable of iron- and sulfur- oxidation, As and Pb resistance, autotroph and lithotroph^28^, which could explain their dominance in sites being nutrient poor or even oligotrophic. Unexpectedly, we detected *Nitrospira*, a most abundant genus (>41%) and *Ralstonia*, a low abundant genus (3%) that possess several functions explaining their adaptability to extreme environments^29^. These functions include carbon fixation, dissimilatory nitrate reduction, metal ion transport, signal transduction for *Nitrospira*, and uptake, efflux, and accumulation of Pb(II)^30^ for *Ralstonia*, which was significantly correlated with Pb contents (*r* = 0.978, *p* < 0.001) (Fig. 4d). Relatively low abundance OTUs (<5%) related to *Sulfurifustis*, *Aciditerrimonas*, and *Bryobacter* were among the most positively linked genera in the bacterial network by cohabiting other genera, whereas *Acidibacillus* and *Alicyclobacillus* were the most negatively linked genera (Fig. 5). Modules involving these genera correlated with pH, Mn, Cu, and Pb (Fig. 5), suggesting that they play a key role in the microbial assemblage.

Among the 21 specific genera detected in the active sites, *Arenimonas* was the most distinctive genus (56% of total abundance; Fig. 2c). This genus, previously isolated from an Fe mine^29^, has not yet been reported in other sites. In our NMMF study, *Arenimonas* was negatively correlated with pH (*r* = −0.998; *p* = 0.002; Fig. 4e). The optimum pH for *Arenimonas* was 8.0^31^, and this bacterium carries genes for alkaline phosphatase, a metalloenzyme with Cd, Zn, and Mg, and a sulfate binding site^31–33^. These functions enable this genus to solidify or mineralize metal(loid)s by biosorption, and to survive harsh environments (average pH 5.0) such as those in the Guangxi active sites.

The presence of low abundant Latescibacteria-related bacteria (Table S6) was noted among the specific genera found in abandoned sites. This uncultured bacterium was also found in hydrocarbon- and chlorinated solvents- contaminated environments^34^, but not previously detected in active or abandoned NMMF sites. Latescibacteria possess the benzylsuccinate synthase (*bss*A) and alkylsuccinate synthase (*ass*A) genes for PAHs and alkanes degradation^35^, which would enable FRs degradation. *Sulfobacillus*, *Acidiferrobacter*, *Acidibacillus*, and *Acidithiobacillus* were also detected (Fig. 2c). These genera are sulfur- and/or iron-oxidizers possessing the arsenic transporter *ars*A and the arsenic efflux pump *ars*B^36,37^, which favor As(V) reduction via a detoxification process under toxic conditions^38^. The *Acidithiobacillus* genus also carries the metal-related genes (Mn efflux), which could be inhibited by the presence of Cu(II)^39,40^.

But how can bacteria endure the extreme NMMFs contaminated by metal(loid)s and FRs? PICRUSt (phylogenetic investigation of communities by reconstruction of unobserved states) offers the possibility to infer the microbial functions from 16S rRNA sequences, based on a full sequenced genome^41^. The NSTI values were 0.14 and 0.18 for active and abandoned tailings respectively (Table S4), indicating that the PICRUSt analysis was robust. The PICRUSt was performed using the classification schemes of COGs (cluster of orthologous groups), to explore the possibility that the collected bacteria developed adaptive metabolically functional capabilities. Category A (RNA processing and modification) showed significant differences between active and abandoned tailings (*p* = 0.04; Table S8), while in Pb-Zn mine tailings and soil environment, category L (involved in replication, recombination and repair) and category G (carbohydrate transport and metabolism) were shown to be statistically overrepresented^22,42^. The RNA processing and modification are known to alter RNA structure-function relationships and various cellular processes^43^. Some COGs related to the detoxification of metal(loid)s and degradation of organics, such as transcriptional regulators (COG0583, COG1309, COG1846, and COG2204), and dehydrogenases (COG1012 and COG1028) were abundant (Fig. S5). Hydrolases are known to favor the degradation of organics^44^, and dehydrogenases such as, ‘oxidoreductases’ are known to be involved in S- or As- oxidation^45^. TP, Fe, As, Cu, and Zn were driving the distribution of most of the COGs involved in transcriptional regulators, dehydrogenases, hydrolase (COG0596), and RNA polymerase (COG 1595) (Fig. S5), confirming that the bacterial communities harbor metabolic pathways to survive in the extreme environment. Based on this information, bacteria with these functions may play a key role at the Guangxi NMMFs sites by coping with the severe conditions by using (i) functional genes (sulfur- and/or iron-oxidizing genes, *bss*A, *ass*A, *ars*A, and *ars*B) in FRs degradation and metal/sulfate binding (or efflux) mechanisms; (ii) intracellular detoxification, and/or (iii) metabolic functional development of the RNA processing and modification under the environmental pressure^37,46,47^. However, to better understand the functions involved in FRs degradation and metal transformation further analyses are required. Mesocosms experiments maintaining soil tailings as close as possible to environmental conditions prevailing in NMMFs would permit to better identification of the involved genes.

Attempts to perform long-term and cost-effective remediation at active and abandoned mine sites can be hampered by the lack of suitable microbial consortia. These consortia should use FRs as a C source, have highly efficient FRs-degradability, and promote precipitation of secondary sulfide minerals that immobilize pollutants. As a possible genetic resource of a co-contaminated environment, determination of uncultured or rarely detected bacterial species and their genetic potential can favor bioremediation. A constructed bacterial consortium, including both installation and colonization of bacterial communities can alleviate toxic chemical stress and maintain the ecological stability. Our findings provide important fundamental data of actual contaminated environments, which were used to identify unique microbial communities and whose future use will reflect a targeted view of cost-effective bioremediation for active and abandoned sites.

## Materials and Methods

### Sampling and geochemical characterization

Sampling sites were located in Guangxi Province, China (107°N, 24°E) (Fig. 1). This area has a subtropical monsoon prevailing climate, with an annual average temperature 20.58 °C. Total average annual rainfall is 1475 mm^48^. Earlier studies showed that higher bacterial community abundance and activity occur during the rainy season^49,50^. Therefore, sampling was performed during June 2016 (typically having relatively high temperatures and rainfall), following a random sampling strategy according to the technical specifications for soil environmental monitoring of the State Environmental Protection Administration (HJ/T 166–2004). The sampling area covered about 1.1 km^2^. Samples (500 g each) were collected in triplicate from four active (sites T_Active_1 to T_Active_4) and nine abandoned (sites T_Aband_1 to T_Aband_9) sites, within the surface layer (0–10 cm) using wood spatulas (Fig. 1). All sites (by confirmation) did not receive amendment or were remediated. At each site, samples were prepared from 3–10 subsamples (depending on each site’s surface area) taken from the uppermost 10 cm of sites. Heterogeneous samples and environment at sites T_Aband_1, T_Aband_2, T_Aband_3, T_Aband_4, and T_Aband_6 were weakly acidic (pH from 5.99 to 6.80), samples T_Aband_5, T_Aband_7, T_Aband_8, T_Active_1, and T_Active_2 were acidic (pH from 2.11 to 2.81), and samples T_Aband_9, T_Active_3, and T_Active_4 had weakly alkaline sites (pH from 7.55 to 7.71) (Table S9). Samples were stored in plastic tubes in a refrigerator at 4 °C, and were transported to the University of Science and Technology Beijing within 2 d of sampling. Triplicate subsamples of each site were then combined (composite samples) and stored at −20 °C for molecular biology analysis. Another aliquot was air-dried and sieved at 100-mesh size (0.149 mm: US standard) for geochemical analyses.

The pH values were determined using a mixture of deionized water and site material, in a water-to-sample ratio of 2.5:1 (v/w). Total organic carbon (TOC) and total nitrogen (TN) were determined using a total organic carbon analyzer TOC-V_CPH_, and a total nitrogen module TNM-1 (Shimadzu), respectively, as described earlier^51^. Total phosphorus standard solutions were prepared for TP soil determinations, using the alkali fusion-Mo-Sb spectrophotometric method (HJ 632–2011). Total concentrations of metals (Cd, Cu, Fe, Mn, Pb, and Zn), metal(loid)s (As and Sb) in the sample extract were obtained using inductively coupled plasma optical emission spectrometry (iCAP 7000 SERIES, Thermo Scientific) after microwave digestion in a 5:3:2 solution of nitric, hydrochloric, and hydrofluoric acids (v/v/v), following Holmström *et al*.^52^. A standard solution of metal(loid)s (GSB 04-1767-2004) (100 μg/mL) was purchased from National Center of Analysis and Testing for Nonferrous Metals and Electronic Materials (NCATN, China), and stored in the dark at 4 °C. The operating conditions were: auxiliary gas flow: 0.5 L/min; plasma gas stabilization time: 10 min; ICP RF power: 1150 W; and pump rate: 45 rpm. Prior to analysis, the inductively coupled plasma optical emission spectrometer (ICP-OES), located in a temperature-controlled laboratory (20 ± 2 °C), was stabilized for a sufficient period before optimization. The limit of detection (LOD) was determined earlier^53^; an Environmental Monitoring-Technical guideline was used for drawing and revising analytical method standards (HJ 168-2010). The individual geochemical properties data, LOD, and recovery of the spiked standard are shown in Table S9. The LOD for metal(loid)s was from 0.10 × 10^−3^ (Zn) to 4.22 × 10^−3^ (Sb) mg/kg. The recovery of the spiked standard was 99.0–103%.

A gas chromatography-mass spectrometer (GC-MS) (Pegasus 4D, LECO Corp) equipped with Restek fused silica chromatography columns, Rxi-5SilMS (30 m × 0.25 mm × 0.25 μm) and Rtx-200 (1.5 m × 0.18 mm × 0.2 μm), was used for analysis of organic compounds in the active and abandoned sites samples (sieved at 100-mesh size), following McGrath *et al*.^54^. The column temperature of the GC was 80 °C (maintained for 2 min), 5 °C/min for up to 300 °C, and maintained for 10 min. The injector temperature was 280 °C. The He carrier gas was high purity (99.999%) with a column flow rate of 1 mL/min. The MS detection system had an electro-impact source (EI) temperature at 250 °C, acquisition frequency of 100 spec/s, and detector voltage at 1650 V.

### DNA extraction and sequencing

DNA was extracted from samples using a SoilGen DNA Kit (CWBio, China) according to the manufacturer’s protocol. To obtain a sufficient DNA concentration for sequencing, 10 g of each sample was used. The purity and concentration of DNA for each sample were analyzed by using NanoDrop2000 (Thermo Fisher Scientific, USA). DNA integrity was determined using 0.8% agarose gel electrophoresis for 5 V/cm for 30 min. PCR amplifications were conducted in triplicate, to amplify the V3-V4 region of the 16S rRNA gene with the 338 F/806 R primer set, where an 8 bp barcode identified the samples^55^. The PCR amplification was performed using TransGenAP221-02 and TransStart Fastpfu DNA Polymerase with 20 μL reaction mixture (including 4 μL of 5× FastPfu Buffer, 2 μL of mM dNTPs, 0.2 μM of each primer, 0.4 μL FastPfu Polymerase, 0.2 μL BSA, 10 ng DNA template, and ultrapure sterilized water). The PCR amplification response parameters were: (a) 1 × (3 min at 95 °C); (b) 28 × (30 s at 95 °C; 30 s at 55 °C; 45 s at 72 °C); and (c) a final extension for 10 min at 72 °C, 10 °C. Sequencing was performed by a commercial facility (Shanghai Majorbio Bio-Pharm Technology Corporation, Shanghai, China) using a MiSeq platform.

Preliminary quality trimming of the MiSeq sequencing were done by FLASH and Trimmomatic 0.33 software, with the restrictions of at least 10 bp of overlap between read pairs and 0.2 allowed mismatches. The sequences assigned to chloroplasts, mitochondria, or Eukaryota were removed in the pretreatment of raw reads. The assembled reads were further denoised by clustering similar sequences with less than 3% dissimilarity using USEARCH (version 7.0 http://drive5.com/uparse/), as well as chimera detection conducted with UCHIME v5.1. Silva (Release128 http://www.arb-silva.de) database bacterial reference alignment assigned the taxonomic ranks for each sequence, with 0.7 confidence score as cutoff ^56^. Sequencing resulted in 490,581 sequences for the bacteria dataset from 13 NMMFs samples (Table S4). The sequencing dataset was normalized for uneven sequencing depth for each sample, and rarefied to the same number of corresponding to the minimum sequencing number (31600 sequences). Operational taxonomic units (OTUs) at a 3% dissimilarity level^57^ were clustered. Sequencing clustering resulted in 1,859 operational taxonomic units (OTUs) with higher taxonomic groups (Table S7). Alpha diversity analysis was performed using Mothur (version v.1.30.1), with a 3% dissimilarity level of OTUs. Three α-diversity indices (normalized for uneven sequencing depth) are presented in Table S4. Good’s coverage data (>99.7 and >99.4% at active and abandoned sites, respectively) indicated that the increase of sequencing depth could only be produced by a small amount of new species.

The accuracy of PICRUSt on the hypersaline microbial mats community is lower than that found on humans; however, this limitation was rectified by including habitat information and the nearest sequenced taxon index (NSTI) of phylogenetic distances to the nearest reference genome^37^. PICRUSt analyses predicted the potential function in each community for functional profiles of bacterial communities. Sequences of 13 NMMFs samples were used to carry out functional annotation tasks, including categorized with COG functional annotation^58^. NSTI characterized the accuracy of PICRUSt, with lower values indicating a higher accuracy^40,59^. Compared with the mean NSTI values of soil (0.17)^40^, the mean NSTI values of active and abandoned sites samples (0.14 and 0.18, respectively) (Table S4) indicated that the genome database could be used to generate the PICRUSt prediction analysis.

### Statistical analyses

Semi-quantitative data of geochemical characteristics including FRs content were obtained from composite samples of 13 NMMFs sites. Due to non-normality, heterogeneity, and outliers of geochemical data, one-variable analysis (Statgraphics statistical software XVI.I) was used to calculate the minimum, lower quartile, median, upper quartile, and the maximum values. The frequency histogram, quantile, scatterplot, and box-and-whisker plot are also presented. To test whether statistically significant differences of quantitative geochemical characterizations existed between active and abandoned sites, we used independent sample *t*-tests with confidence interval of 0.95 (SPSS statistical software version 21).

The similarity of active and abandoned sites samples was assessed by calculating Euclidean distances based on a hierarchical classification analysis (Fig. S2). Canonical correlation analysis (CCA) for 16S rRNA sequencing data was used to reveal similarity of bacterial diversity in NMMFs sites according to site geochemistry (i.e., pH, TN, TOC, TP, and content of metal(loid)s) (Fig. S2). As well, CCA analysis used the envfit function to calculate the *p*-value of correlation between each geochemical factor with overall OTUs. Bacterial community similarity and differences are presented by Venn analysis using R v.3.0.1 software. Circos-0.67-7 was used to explore the proportion of dominant species for each sample. To test the differences of bacterial communities between sites, Wilcoxon rank-sum test was used based on false discovery rate (FDR) multiple check correction and 95% confidence intervals. To investigate the differences of bacterial community composition between active and abandoned sites in the present study and at other mine sites differing in the type and geographic locations, the percentage of bacterial communities were obtained from previous studies, i.e., at Pb-Zn mine^22,23^, REE mine^24^, and chlor-alkali tailings^25^. Differences of geochemical factors, and diversity of bacterial communities between these sites were also analyzed by ANOVA (*p* < 0.05) and independent sample *t*-test (95% confidence intervals) using SPSS software. Network analysis was used to reflect the genera correlation between active and abandoned sites with a 0.5 threshold of correlation coefficient. After module detection, each module was represented by network correlation shared values of abundance profile by using modularity analysis. Modules related to the top 10 high degree genera and microbial diversity response to geochemical factors were analyzed by Monte-Carlo tests (with Pearson correlation test based on 9999 replicates) and biota–environmental matching (BioEnv) analysis. The BioEnv procedure was conducted with metric “Euclidean” and Spearman’s rank correlation (based on Bray-Curtis dissimilarities measures) using R language vegan package. Correlation analysis assessed relationships between microbial and site geochemistry, using the Pearson *t*-test. Significant differences between COGs function classification in active and abandoned sites based on 16S rRNA sequencing reads were tested, using the independent sample *t*-tests (95% confidence intervals). The COGs functional variation related to geochemical characterization was explored by CCA, using distance matrices and a permutation test with pseudo-*F* ratios.

## Electronic supplementary material

Supplementary Materials

Supplementary Table S7
