# Supplementary material for: China’s most typical nonferrous organic-metal facilities own specific microbial communities
Source: Sci Rep. 2018 Aug 22;8:12570. doi: 10.1038/s41598-018-30519-1 (PMC6105654; doi:10.1038/s41598-018-30519-1)
Supplement: Supplementary file 1 — Supplementary Materials [file 41598_2018_30519_MOESM1_ESM.doc]

**Supplementary Materials**

**China’s most typical nonferrous organic-metal facilities own specific microbial communities**

**Jian-li Liu ^1^, Jun Yao ^2^, Fei Wang ^1^, Wen Ni ^3^, Xing-yu Liu ^4^, Geoffrey Sunahara ^5^, Robert Duran ^6^, Gyozo Jordan ^7^, Karen A Hudson-Edwards ^8^, Lena Alakangas ^9^, Tatjana Solevic Knudsen ^10^, Xiao-zhe Zhu ^1^, Yi-yue Zhang ^1^, Zi-fu Li ^1^**

^1^ School of Energy and Environmental Engineering, University of Science and Technology Beijing, Beijing 100083, China

^2^ School of Water Resource and Environmental Engineering, China University of Geosciences (Beijing) 100083, China

^3^ School of Civil and Resource Engineering, University of Science and Technology Beijing, Beijing 100083, China

^4^ Gen Res Inst Nonferrous Met, Natl Engn Lab Biohydromet, Beijing 100088, China

^5^ Department of Natural Resource Sciences, McGill University, Montreal, Quebec, H9X3V9, Canada

^6^ Equipe Environnement et Microbiologie, MELODY group, Université de Pau et des Pays de l’Adour, IPREM UMR CNRS 5254, BP 1155, 64013 Pau Cedex, France

^7^ Department of Applied Chemistry, Szent István University, Villányi út 35-43, 1118 Budapest, Hungary

^8^ Environment & Sustainability Institute and Camborne School of Mines, University of Exeter

^9^ Lule University of Technology, -971 87 Lule1, Sweden

^10^ Institute of Chemistry, Technology and Metallurgy, University of Belgrade, Njegoseva 12, POBox 473, 11001 Belgrade, Serbia. 

* J Yao (yaojun@cugb.edu.cn) and ZF Li ([zifulee@aliyun.com](mailto:zifulee@aliyun.com))

**Running title:** China’s most typical nonferrous organic-metal facilities

**Word count:** 3930 (excluding Abstract, References and Figure legends)


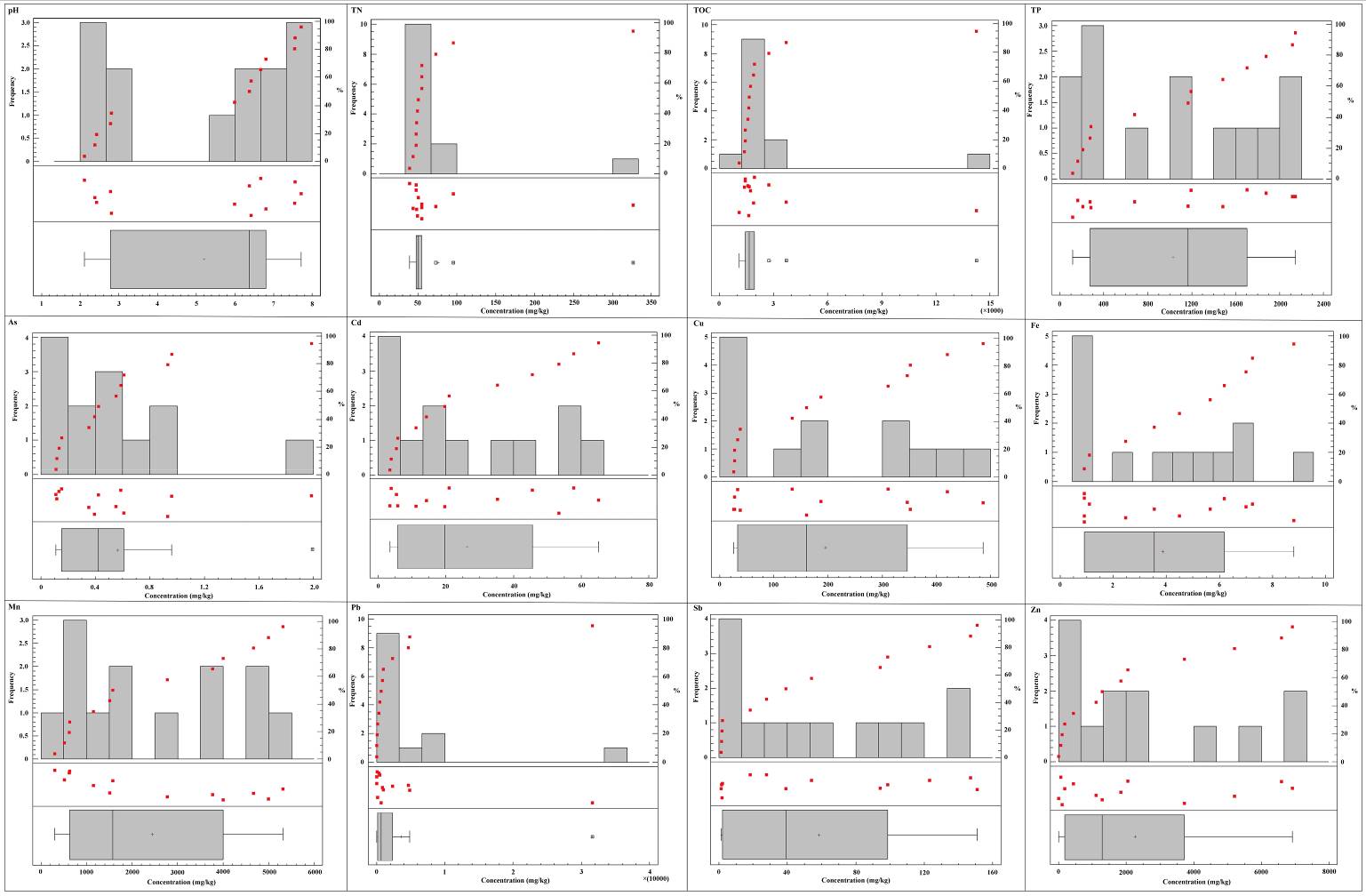


**Supplementary Figure S1: Distribution plots (frequency histogram, quantile plot, scatterplot and box-and-whisker plot) of geochemical properties.** The left Y axis denotes frequency histogram, and right Y axis denotes sample percentiles for each geochemical property. The box-and-whisker plots summarize the minimum, median, and maximum quartiles, and the presence of outliers is indicated. TP, Cd, Cu, Fe, Mn, Pb, and Zn were homogeneously distributed as evidenced by the histogram having no outlying values. The distributions of TN, TOC, As, and Pb were heterogeneous with only one outliner at different sites.


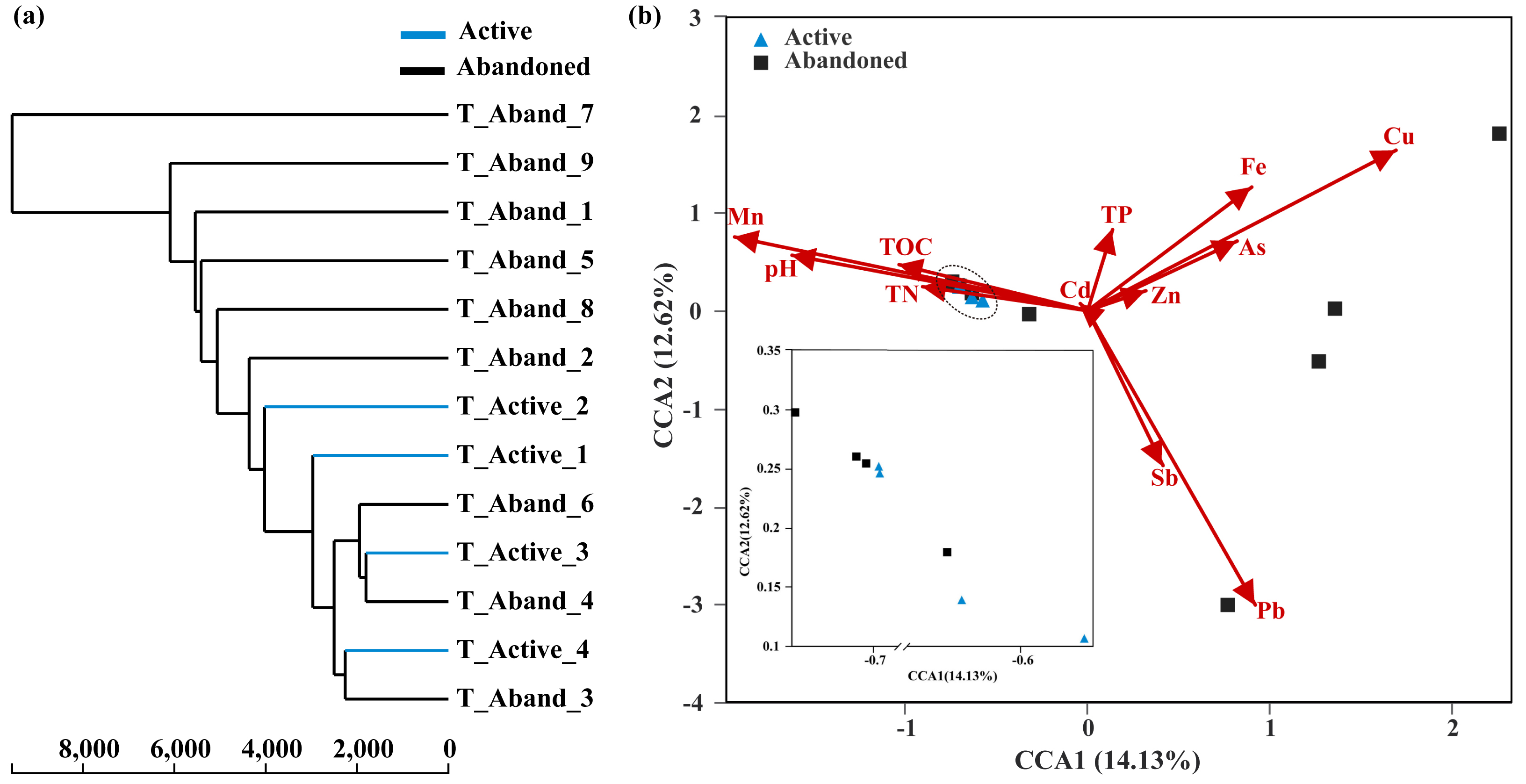


**Supplementary Figure S2:** **Hierarchical classification analysis of Euclidean distances between the active and abandoned sites (left panel). Canonical correspondence analysis (CCA) plots for bacterial communities based on OTUs (right panel).** The dotted circle in the right panel (b) was magnified as shown in the inset. The detailed data for correlation and *p*-values are shown in Supplementary Table S6.


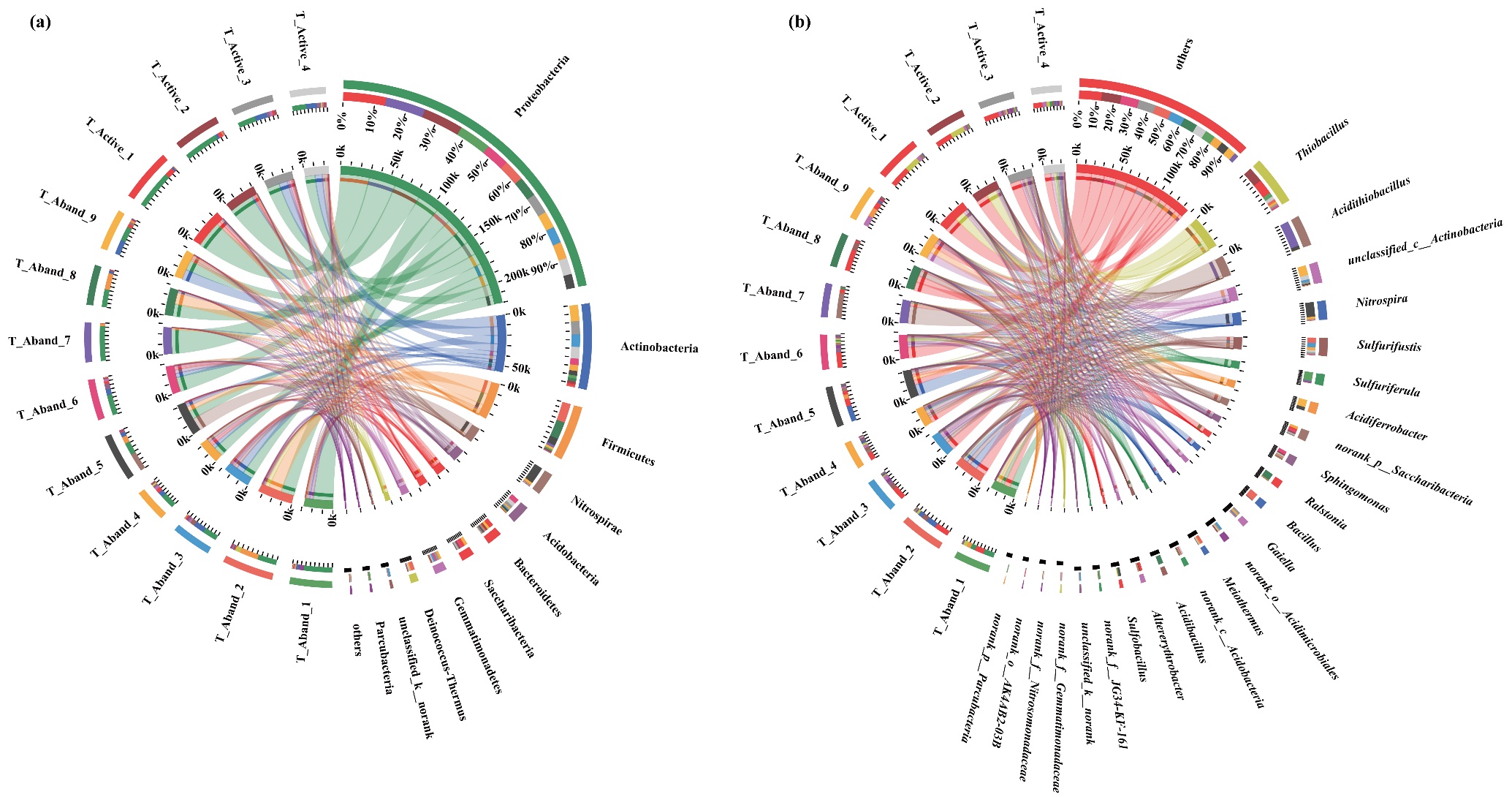


**Supplementary Figure S3: Composition and relative abundance of dominant species in each sample and comparison of bacterial communities in 13 NMMFs at phylum (left panel) and genus (right panel) levels.** Left side semicircle indicates the composition of species abundance in “active” and “abandoned” facility site samples. Two pairs of concentric circles are shown. The outer broken circle of each pair denotes the color-coded sample. The inner broken circle of the outer shell denotes the relative bacterial abundance within each sample. The inner broken circle of inner shell maps the abundance of bacteria to the species identification.


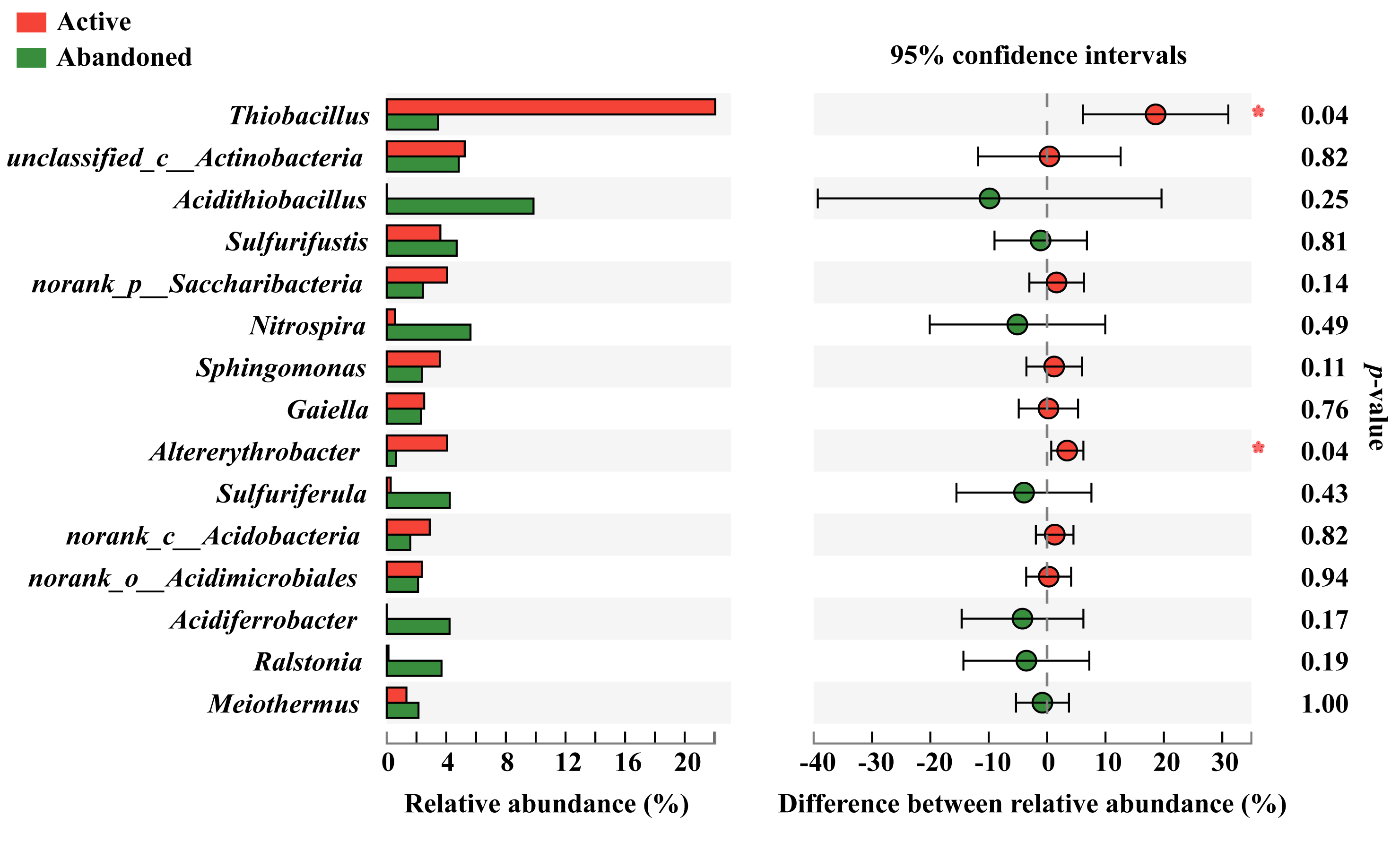


**Supplementary Figure S4: Wilcoxon rank-sum test bar plot on genus level based on 16S rRNA sequencing reads in both active and abandoned sites.** Colors are according to facility type, plotted by the relative abundance of a genera in the sample on the horizontal axis, and genus on the vertical axis. The top 15 genera were used for analysis. * 0.01 < *p* ≤ 0.05; ** 0.001 < *p* ≤ 0.01; *** *p* ≤ 0.001

**
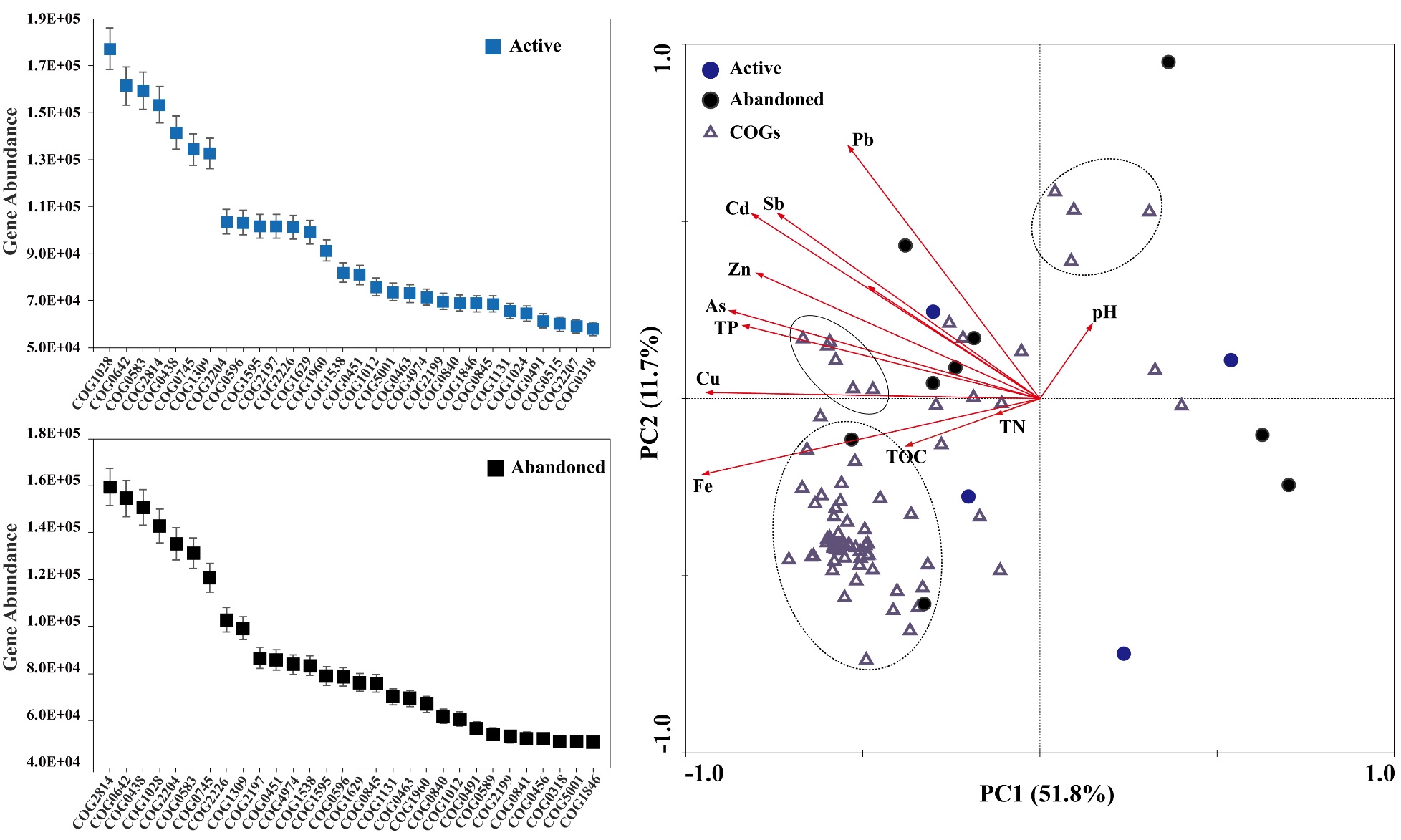
**

**Supplementary Figure S5: Cluster of Orthologous Groups of proteins (COGs) function classification in active and abandoned sites based on 16S rRNA sequencing reads (left panel).** **Principal Components Analysis (PCA) for geochemistry and COGs function classification based on 16S rRNA sequencing reads in both active and abandoned sites (right panel).** The top 30 and 100 COGs were used for analysis. The dotted circles (in right panel) represent the clustering of COGs.

**Supplementary Table S1:** Distribution of organic compounds in 13 NMMFs study samples using gas chromatography-mass spectrometry.

**Supplementary Table S2: Se**lected geochemical properties of 13 NMMFs study sites.

|  | **BG value** | **Min** | **LQ** | **Med** | **UQ** | **Max** | **Avg** | **SE** | **SD** | ***p*-value** | ***t*-value** |
| --- | --- | --- | --- | --- | --- | --- | --- | --- | --- | --- | --- |
| pH | 6.7 | 2.1 | 2.8 | 6.4 | 6.8 | 7.7 | 5.2 | 0.6 | 2.3 | 0.95 | -0.07 |
| TN (mg/kg) | 1270 | 39 | 48 | 50 | 55 | 327 | 76 | 21 | 77 | 0.50 | -0.71 |
| TOC (mg/kg) | 23600 | 169 | 538 | 762 | 1080 | 14200 | 1990 | 1040 | 3740 | 0.68 | -0.43 |
| TP (mg/kg) | 5300 | 119 | 276 | 1168 | 1703 | 2143 | 1030 | 217 | 782 | 0.67 | -0.45 |
| As (mg/kg) | 11 | 105 | 509 | 3240 | 5090 | 18900 | 4620 | 1430 | 5140 | 0.12 | -1.68 |
| Cd (mg/kg) | 0.097 | 0.57 | 2.83 | 16.7 | 42.6 | 62.2 | 23.3 | 6.2 | 22.4 | 0.54 | -0.67 |
| Cu (mg/kg) | 22.6 | 5.8 | 13.0 | 140.0 | 326.0 | 466.0 | 176.0 | 47.0 | 168.0 | 0.83 | -0.22 |
| Fe (mg/kg) | 2.94 | 0.00 | 0.00 | 29100 | 580 | 86900 | 32400 | 8780 | 31700 | 0.92 | -0.10 |
| Mn (mg/kg) | 583 | 0.00 | 333 | 1280 | 3700 | 5010 | 2150 | 520 | 1880 | 0.92 | -0.11 |
| Pb (mg/kg) | 26 | 14.2 | 128 | 646 | 2320 | 31600 | 3620 | 2380 | 8580 | 0.26 | -1.21 |
| Sb (mg/kg) | 1.2 | 1.3 | 2 | 39 | 99 | 151 | 59 | 16 | 57 | 0.01* | -2.91 |
| Zn (mg/kg) | 74.2 | 0.00 | 190 | 1300 | 3720 | 6920 | 2270 | 697 | 2520 | 0.73 | -0.36 |

BG, Background soil values of China; Min, minimum; LQ, lower quartile; Med, median; UQ, upper quartile; Max, maximum; Avg, average; SE, standard error; SD, standard deviation; TN, total nitrogen; TOC, total organic carbon; TP, total phosphorus; * significantly different at *p* < 0.05

**Supplementary Table S3:** Summary of geochemical properties of active and abandoned sites.

|  |  | **BG value** | **Min** | **LQ** | **Med** | **UQ** | **Max** | **Avg** | **SE** | **SD** | ***p*-value** |
| --- | --- | --- | --- | --- | --- | --- | --- | --- | --- | --- | --- |
| pH | Active | 6.7 | 2.42 | 2.62 | 5.18 | 7.63 | 7.71 | 5.12 | 1.45 | 2.90 | 0.95 |
|  | Aband |  | 2.11 | 2.78 | 6.38 | 6.67 | 7.56 | 5.23 | 0.72 | 2.15 |  |
| TN (mg/kg) | Active | 1270 | 39.0 | 44.0 | 52.0 | 75.1 | 95.4 | 59.6 | 12.4 | 24.8 | 0.50 |
|  | Aband |  | 43.7 | 47.5 | 50.3 | 54.8 | 327 | 82.9 | 30.6 | 91.9 |  |
| TOC (mg/kg) | Active | 23600 | 538 | 604 | 1290 | 2430 | 2950 | 1520 | 569 | 1140 | 0.68 |
|  | Aband |  | 169 | 526 | 762 | 1020 | 14200 | 2200 | 1500 | 4510 |  |
| TP (mg/kg) | Active | 5300 | 119 | 163 | 847 | 1590 | 1700 | 879 | 416 | 832 | 0.67 |
|  | Aband |  | 164 | 282 | 1170 | 1880 | 2140 | 1100 | 267 | 801 |  |
| As (mg/kg) | Active | 11.2 | 105 | 225 | 1790 | 3880 | 4520 | 2050 | 1090 | 2180 | 0.12 |
|  | Aband |  | 176 | 2510 | 4870 | 8320 | 18900 | 5760 | 1920 | 5750 |  |
| Cd (mg/kg) | Active | 0.097 | 0.6 | 1.5 | 5.4 | 31.6 | 54.8 | 16.6 | 12.9 | 25.7 | 0.54 |
|  | Aband |  | 1.0 | 11.3 | 18.0 | 42.6 | 62.2 | 26.4 | 7.22 | 21.7 |  |
| Cu (mg/kg) | Active | 22.6 | 7.7 | 7.7 | 149 | 311 | 332 | 160 | 88.1 | 176 | 0.83 |
|  | Aband |  | 5.8 | 18.1 | 140 | 326 | 466 | 183 | 58.0 | 174 |  |
| Fe (mg/kg) | Active | 2.94 | 0.00 | 1040 | 27100 | 60900 | 69700 | 31000 | 17700 | 35300 | 0.92 |
|  | Aband |  | 0.00 | 0.00 | 29100 | 58100 | 86900 | 33100 | 10700 | 32200 |  |
| Mn (mg/kg) | Active | 583 | 211 | 531 | 1660 | 3580 | 4690 | 2060 | 999 | 2000 | 0.92 |
|  | Aband |  | 0.00 | 333 | 1280 | 3700 | 5010 | 2190 | 647 | 1940 |  |
| Pb (mg/kg) | Active | 26 | 14.2 | 59.7 | 298 | 1410 | 2320 | 733 | 540 | 1080 | 0.26 |
|  | Aband |  | 19.4 | 330 | 828 | 4690 | 31600 | 4900 | 3400 | 10200 |  |
| Sb (mg/kg) | Active | 1.20 | 1.25 | 1.53 | 10.1 | 28.8 | 39.3 | 15.2 | 9.0 | 17.9 | 0.01* |
|  | Aband |  | 1.5 | 28.0 | 94.4 | 123 | 151 | 77.8 | 19.5 | 58.6 |  |
| Zn (mg/kg) | Active | 74.2 | 67.0 | 128.5 | 317.2 | 3520 | 6600 | 1820 | 1590 | 3190 | 0.73 |
|  | Aband |  | 0.00 | 1110 | 1850 | 3720 | 6920 | 2470 | 784 | 2350 |  |

BG, Background soil values of China; Min, minimum; LQ, lower quartile; Md, median; UQ, upper quartile; Max, maximum; SE, standard error; SD, standard deviation; TN, total nitrogen; TOC, total organic carbon; TP, total phosphorus; * significantly different at *p* < 0.05

**Supplementary Table S4: Alpha-diversity indices of bacterial communities of the 13 NMMFs sites samples (active and abandoned sites).** Shannon and ace diversity indexes were determined based on the similarity of 97 % (OTU_97_) from16S rRNA gene sequencing data.

NSTI, nearest sequenced taxon index.

**Supplementary Table S5: Correlations between the bacterial α-diversities and** **the geochemical properties using Spearman and Monte-Carlo tests.**

TN, total nitrogen; TOC, total organic carbon; TP, total phosphorus; Heterocyclic, Heterocyclic Compounds; rS, Spearman correlation with BioEnv test; rM, Monte-Carlo test correlation; * best model has 3 parameters, pH, Cu and Pb with best correlation to α-diversity indexes; *p*-values < 0.05 were considered statistically significant.

**Supplementary Table S6: The detailed data for correlation and *p*-values of are Canonical correspondence analysis (CCA).**

|  | **CCA1** | **CCA2** | **r** | ***p*-values** |
| --- | --- | --- | --- | --- |
| **pH** | -0.944 | 0.3298 | 0.2395 | 0.276 |
| **TN** | -0.9652 | 0.2617 | 0.0714 | 0.404 |
| **TOC** | -0.9103 | 0.414 | 0.1048 | 0.307 |
| **TP** | 0.1633 | 0.9866 | 0.0571 | 0.794 |
| **As** | 0.7555 | 0.6551 | 0.0957 | 0.492 |
| **Cd** | -0.4844 | 0.8749 | 0.0006 | 0.997 |
| **Cu** | 0.7181 | 0.696 | 0.4506 | 0.021 |
| **Fe** | 0.5805 | 0.8143 | 0.1954 | 0.344 |
| **Mn** | -0.9319 | 0.3627 | 0.3504 | 0.077 |
| **Pb** | 0.2924 | -0.9563 | 0.8037 | 0.025 |
| **Sb** | 0.2528 | -0.9675 | 0.2175 | 0.324 |
| **Zn** | 0.8433 | 0.5374 | 0.0116 | 0.936 |

**Supplementary Table S7: The average OTU distribution for 16S rDNA sequences for both active and abandoned tailings sites.** The table has been listed as a separate worksheet file.

**Supplementary Table S8: COGs functional classification differences between active and abandoned sites.**

Mean Difference: the average data of COGs functional classification between active and abandoned sites,

The COGs functional description of general category letter associations:

(ⅰ) Information Storage and Processing

A: RNA processing and modification,

B: chromatin structure and dynamics,

J: translation, ribosomal structure, and biogenesis,

K: transcription,

L: replication, recombination, and repair,

(ⅱ) Metabolism

C: energy production and conversion,

E: amino acid transport and metabolism,

F: nucleotide transport and metabolism,

G: carbohydrate transport and metabolism,

H: coenzyme transport and metabolism,

I: lipid transport and metabolism,

P: inorganic ion transport and metabolism,

Q: secondary metabolites biosynthesis, transport, and catabolism,

(ⅲ) Cellular Processes and Signaling

D: cell cycle control, cell division, chromosome partitioning,

M: cell wall/membrane/envelope biogenesis,

N: cell motility,

O: posttranslational modification, protein turnover, chaperones,

T: signal transduction mechanisms,

U: intracellular trafficking, secretion, and vesicular transport,

V: defense mechanisms,

W: extracellular structures,

Z: cytoskeleton.

(ⅳ) Poorly Characterized

R: general function prediction only,

S: unknown function.

**Supplementary Table S9: Characteristics of geochemical factors and limit of detection of metal(loid)s in both active and abandoned sites.**

Sample ID, the name of sampling site; TOC, total organic carbon; TN, total nitrogen; TP, total phosphorus; LOD: limit of detection
